# Electricity in Congenital Deafness

**Published:** 1858-12

**Authors:** 


### ELECTRICITY IN CONGENITAL DEAFNESS.

In the Bulletin geniral de Theraputique (Oct. 1858.) M. Duchenue (de Bologne) publishes the favorable result obtained by electricity in a case of congenital deafness. The subject was a boy eight years old, who had been deaf from his birth, and consequently dumb. The machine used by M. Duchenue was appropriate to the delicateness of the organ to be acted upon.

The circuit was made by first filling the external ear with water, and then introducing into this fluid one of the conductors, so arranged by a tube of some non-conducting material, as to prevent its coming in contact either with the tympanum or walls of the external ear, the circuit then completed by placing the other conductor upon the mastoid process. M. Duchenue commenced the experiment without any hope of a favorable result, and was agreeably surprised to find during the fourth application that he was able to detect sounds. After the fifth and sixth application he could hear sufficiently distinct to attempt the imitation of sounds. This education was commenced by pronouncing the vowels, and after the seventh or eighth sitting he was in a few days able to pronounce them all, and also to pronounce the words Pa, Ma, &c., and understand their meaning.

M. Duchenue remarks that his intelligence, habits, disposition &c., underwent a rapid and complete transformation as the improvement continued.

The child has been under treatment for eighteen or twenty months, the applications made at long intervals and each marked with benefit. So great has been the improvement that he is now receiving his education in the ordinary way. The treatment will be continued.

This is certainly a very interesting case, and proves that congenital deafness depends, in some cases at least, alone upon a fault in the functions of the nerves of the part, as it has long ago been demonstrated that in a number of cases there can be no apparent lesion found in the construction of the auditory apparatus. We shall look with great interest to M. Duchenue's experiments.

